# The Hospitals and Surgeons of Paris

**Published:** 1844-09

**Authors:** 


Art. IV.—*The Hospitals and Surgeons of Paris.* An Historical and Statistical account of the Civil Hospitals of Paris; with Miscellaneous Information, and Biographical Notices of some of the most eminent of the living Parisian Surgeons. By F. Campbell Stewart, M.D. N. York: J. & H. G. Langley. Phila.: Carey & Hart. pp. 432, 8vo: 1843.

Dr. Stewart spent about four years in what a distinguished writer calls "the seat of all dissoluteness," Paris, and, if we are correctly informed, under circumstances peculiarly favorable to professional improvement and observation. He seems to have been industriously occupied, and this publication, which has been before the public some time, is the first fruits of his industry.

Dr. Stewart speaks of the civil hospitals only, and presents, without doubt, the best and most concise account of them within the reach of the American reader. One cannot rise from a perusal of the book without a sentiment of admiration for a Government, which, whatever may be its faults, provides such noble charities for its subjects. It is not merely a wise economy (for such there undoubtedly is) that has dictated them, but something far higher and nobler. The foundation of the present system dates from the Republic and the Empire; until that period, the French hospitals were in a most wretched condition. Much of their present efficiency is due to one whose ambition was not more grasping than his intellect was comprehensive. Napoleon educed order and system from the civil chaos of the Revolution, and infused them into every part of his Government. Something of course has been done since his time, but to him, we believe, the French people are indebted for many of the most admirable features in their system of hospitals.

The administration of the civil hospitals is conducted by 1st. a *General Council*, consisting of seventeen members; 2d. an *Administrative Committee*, composed of five members of the general council; 3d. a *Banker* and a *General Secretary*. The general council forms a part of the Home Department of the government, and communicates with the Minister of the Interior.

The hospitals are divided into three classes: *General Hospitals*, *Special Hospitals*, and *Hôspices*, or *Alms-houses.* Of all these together there are thirty-six. Each is provided with a *Director; Surgeons* and *Physicians* in proportion to the number of beds, allowing about 60 beds to each; an *Apothecary; Internes*, or Resident Physicians, and *Externes*, or "Dressers;" *Students of Pharmacy; Sisters of Charity;* a *Priest;* a *Steward;* and male and female *domestics.*

Belonging to the general administration is the *Bureau Central*, or office where patients apply for admission into the hospitals. A certain mumber of medical men are attached to it, chosen by "concours," and it is from this body that the hospital physicians and surgeons are selected. Then there are the "*Pharmacie Central*," or general laboratory, for the preparation of all the medicines used in the hospitals and prisons; the *Bakery*, an immense establishment; and the *Wine Vaults.*

The reader will see what a vast system it is, and yet the most perfect order and economy reign throughout. Our author gives an historical sketch of each of the institutions, with minute information of everything relating to them that would interest the student or professional man. We cannot, without doing him injustice, attempt to follow him through these various details; for all which, and much more than we have named, we refer the reader to the book itself.

The average population of the hospitals is about 20,000. During 1840, the whole number of patients treated in the hospitals was 83,643. Of these 61,883 were medical patients, and 21,760 surgical patients. What a field for study!

The annual expenses of all these establishments exceed *twelve millions* of francs. The revenues exceed this amount, so that there remains, at the end of almost every year, a balance unexpended. The revenues are derived in great part from taxes on theatres and places of public amusment; allowances are also made by the city, and by the department in which the city is situated. Another source is indicated in the following extract:

"A large amount is likewise derived from the "*Mont de*

*Piété*," as it is called, or by pawn-broking. All the pawnbroking in Paris is conducted by agents of the hospital administration, and the profits derived from this source go to the general hospital fund; articles pledged may be redeemed at any time within twelve months, on the payment of three-quarters per cent. a month on the amount advanced; if not called for by the expiration of the year, they are disposed of at public auction, and the surplus over the amount advanced, is retained, during three years, to the credit of the depositor; if not withdrawn prior to the expiration of this term, the surplus is passed into the treasury of the hospitals; occasionally very large sums are derived from this source."

One of the most interesting institutions that the author speaks of, is the Veterinary School and hospital at Alfort. This, like all similar establishments, is under the control of the Minister of Public Instruction and Agriculture. The term of study is four years, and the course of instruction is complete on all the branches likely to prove useful in the life of a veterinary surgeon. After describing the *operating-room*, Dr. Stewart says:

"Two days of each week, Monday and Thursday, are set aside as operating-days, and old, broken-down horses are provided *by the Government* (the department of Instruction and Agriculture) for the barbarous purpose of being *dissected alive.* Many of the operations performed are wholly useless, and the cruelty exercised by the young students engaged in them unpardonable and disgraceful. Ten or a dozen horses, which are purchased at about twenty francs a head, are provided on each of the indicated days; they are thrown down and tied with all their limbs together, and a tourniquet applied to the under lip (to keep their heads down and hold them still) at five o'clock in the morning, from which time until five in the afternoon they are subjected to all the mutilations that the young men choose to practise upon them. Each horse has to undergo *sixty-four* operations, after which, should he be still breathing, an end is put to his miseries, and he is cut up for the use of the menagerie at the Garden of Plants. * * *

"When thrown, one of the young men holds the head of the trembling and groaning animal tightly fixed to the ground with the tourniquet, while a dozen others cut and hack at him in the most disgustingly cruel manner; some engaged in extirpating his ears, and others his eyes; one taking up arteries, and another amputating his tail and limbs, and indeed subjecting him to every operation that can be, as well as those that cannot be, advantageously practised on a wounded horse."

A chapter is devoted to *Medical Instruction in France*, *the condition of the profession* there, and *the advantages of studying at Paris.* He thinks the chief inducements to resort to that city for acquiring a medical education are the opportunities presented for studying *Anatomy*, healthy and morbid, human and comparative, *Obstetrics* and *Diseases of Children*, *Diagnosis*, *Diseases of the Skin* and *of the Eye*, *Practical Chemistry*, *Operative Surgery*, *Orthopedy*, *and Botany.* He says nothing in favor of French *practice;* on the contrary, he believes "our own systems of treatment are, in most respects, far superior to those pursued by the majority of the French faculty."

Dr. S. gives a highly interesting account of the Schools of Medicine and Pharmacy, and the courses of instruction in each; also of the Private Courses, Dissecting-Rooms, Libraries, Museums, Cabinets, Academies, Medical Societies, and Botanical Gardens. Under the head of *Miscellaneous Information*, the student will find every thing necessary for him to know as to the expenses and mode of living at Paris, even to the matter of *conveyances* and their prices.

Part II is devoted to biographical sketches of some the most distinguished living surgeons of Paris, and is one of the most attractive portions of the book. We shall confine our notice of it to some extracts from the biography of Velpeau. His name occurs so frequently in books and periodicals that the reader certainly feels desirous to know something of his history; and the author's account of him differs somewhat from that given in a certain book of travels by a distinguished surgeon of this country. We do not mean the one who sacrificed a cock* at the grove of Æsculapius, but the

* This most ridiculous affair has been narrated to us by an eye-witness. The High-Priest, or "Great American Surgeon" (this he caused himself to be heralded on approaching a village), it will be remembered, not only disturbed the sphered shade of the old bald-headed Greek, but thought to tickle his own palate by something of a *gustatio* from that same cock. It proved to be anything but "fat capon;" our informant says it was the "leanest, toughest, blackest, old cock that ever saluted the grey-dawn."

one who tells of all the great men and *all the fine horses* that he saw in his *rambles.*

"Alfred-Armand-Louis-Marie Velpeau is the son of a blacksmith. He was born on the 18th of May, 1795, at the village of Brêche, situated on the side of the main road to Tours, and distant eight leagues from that populous town. His parents were poor, but honest, kind-hearted people, divested of all feelings of ambition, as is evinced from the fact of their having destined their son to follow the laborious and unprofitable trade, from the exercise of which they derived their own support. Thus as soon as he was able to run about and use his limbs, young Alfred's services were required for attending on the forge and keeping his father's bellows in play. He is said to have been of a quiet, reserved disposition, rarely, if ever, indulging in the sports and amusements of his companions, but occupying all his hours of play, and every moment that was allowed him from his irksome task, in endeavoring to learn to read.

"It so happened that, though no scholar himself, and not at all given to study, the elder Velpeau possessed the most extensive library that his village contained, and it was composed of *three works*, old, worm-eaten, thumbed, and dirty. They were a treatise of *Hippicratie*, the *Rustic Mason*, and the *Poor People's Physician.* With the assistance of these books alone, and an occasional lesson from some well-disposed friend, young Alfred was soon enabled to read. This success increased his ambition, and he determined to persevere and gain knowledge from every source that presented itself to him. Not being able to get at any new books, he occupied himself in committing to memory the three just cited, so that by the time he was ten years old he had gotten every line of them by heart, and he now began to practise himself in writing, and soon became capable of keeping the small scores of the farriery. It appears that his first predilection for medicine was derived from his study of the receipts and directions contained in one of the works above named; and this predilection shortly evinced itself in a decided manner, for no sooner had he acquired a knowledge of the virtues of some of the remedies in common use, than he commenced their application. It seems that he often amused himself by prescribing for the trifling maladies, cut fingers, bruises, &c., of his companions; and so successful was he in their treatment, that the urchins always sought him out on the occurrence of the slightest accident. An anecdote is related of his having cured himself of a troublesome ulcer of the leg, for which he had been attended for a long time by a country doctor, and thus accidentally brought himself into notice, for from that period he was regarded and looked up to as the village oracle, on all medical and surgical subjects."

At this time Velpeau received some instruction from a curate, who came to reside at the village and who gave gratuitous instruction to the children of his flock. A warm attachment sprung up between teacher and pupil, but unfortunately the curate died after four month's residence.

"When sixteen years of age, an unlucky accident occurred, which promised at first to destroy at the same time his young professional reputation, and his hopes of future success and advancement. He had undertaken to prescribe for a young girl, who was ill, and administered a dose of black hellebore, which produced the most alarming symptoms, and jeopardized the life of his young patient. As soon as the extent of the danger was known, a messenger was despatched for a skilful physician, whose residence was at some distance, and during the time intervening until his arrival, the innocent cause of all the difficulty remained in a corner of the sick-room, appearing the picture of despair, and watching anxiously the progress of events, uncertain as to what consequences might ensue to himself. At last the physician arrived, and by his judicious management was able to restore the little sufferer, and allay the immediate apprehensions of its anxious parents. Before his departure, this physician sought out Velpeau, whose history, as he had heard it from the messenger by the way, interested him, and after conversing with him for some time, and noting his enthusiasm for medicine, advised him to prosecute his studies on all occasions, and promised to recommend him to the notice of a wealthy proprietor of the neighborhood, He kept his word, and gave so favorable an account of the young man to his friend, as to induce that gentleman to grant him the privilege of prosecuting his studies under his roof, and with his own children, directing their preceptor to afford him every facility for acquiring knowledge."

Velpeau's progress was so rapid that in a few months he acquired a familiar knowledge of the classics, and was pronounced an excellent Latin scholar. He now set off for Tours, with a small bundle of clothes, a week's provision, and thirty-seven francs in his pocket.

"On his arrival at Tours, his first care was to seek out a cheap lodging, which, after a long search and much trouble, he succeeded in obtaining, nearly if not wholly rent-free, in the garret of an old and dilapidated building.

"During the early part of his sojourn at Tours, he lived on a scanty supply of coarse bread and cheese, which was sent to him once a week by his thoughtful mother; indeed he has often said, that without this fare, poor as it was, he should have had nothing to eat, or been obliged to resort to the unpleasant expedient of seeking charity. The whole of his time, during the day, was passed at the hospital, in attending to the patients and in dissecting, and the greater part of the night was occupied in close study in his small room.

"He appears to have been fortunate in making friends, and amongst them he was able to rank the excellent surgeon of the Hospital of Tours, Monsieur Bretonneau; this gentleman was so much pleased with his great application, and unremitting attention to the patients, that he procured for him the remission of the usual quarterly payment of twenty francs exacted from students attending the hospital. This was of great assistance to Velpeau, as the money thus saved enabled him to provide some few necessaries, and one or two good books.

"The attention of the hospital authorities having been thus directed to him by their chief surgeon, they continued to note his conduct, with which they were so well satisfied as soon to accord him the place of Interne, as a reward for his kindness to the sick, and for the very essential services which he was then capable of rendering to the institution over which they presided. Previous to this, however, and after only fifteen months' study, feeling himself prepared for it, he underwent his examination for the diploma of "Officier de Santé," which he obtained without difficulty, gaining his license with high encomiums from his examiners, and the warm congratulations of his friends."

He was not satisfied with thus gratifying the highest wishes of his parents; his ambition was aroused and he resolved to attain something still higher.

"He became first assistant to Monsieur Bretonneau, and attended with him to his private practice at such times as his other duties would permit. He gradually became known, and began to get a little practice for himself, the receipts from which, together with the two hundred francs allowed him annually by the hospital, enabled him to procure more comforts and live better than he had hitherto done; it did not induce him, however, to relax his exertions, for he continued still to devote every leisure moment to study."

At twenty-five, he fell in love—became engaged to a lady who, though far above him in society, and possessed of some wealth, professed for him the warmest attachment. But—she jilted him. We shall let Dr. S. tell the story, especially as his fling at the sex creates a suspicion that twenty-five was a critical period in his own life.

"One of those unaccountable freaks of woman's fancy, which are of but too frequent occurrence to be creditable to the sex, came, however, in the way of his expected happiness, and caused the lady to dismiss her lover, without assigning any reason for so doing. Mortified and provoked at such treatment, he at once determined to leave the place of her abode and his fickle mistress behind him. * * * After hesitating for some time time, and yet remaining in a state of uncertainty, it is believed that he only determined to seek Paris by the advice and at the earnest solicitation of his friend M. Bretonneau, who kindly provided him with a letter of introduction and recommendation to the excellent Dr. Jules Cloquet, with whom he was intimately acquainted, and who pressed on him the acceptance of a loan of ten Napoleons, (about $40.)"

With a few clothes, and four hundred francs in money, he set out for Paris. On arriving there, he rented a small room at *seven francs* a month, and immediately made his way to the hospitals, between which and the public libraries he spent his whole time. He lived on coarse ammunition bread and water, and economised so closely that after the lapse of three months he had spent only 100 francs. By the advice of Cloquet, who had manifested considerable interest in him, he now entered the "concours" and gained a situation in the hospital St. Louis. Here he remained three years, gained the anatomical and physiological prizes for the first year, and delivered lectures on anatomy and surgery to the junior students. Next he was for some time private assistant to Dr. Bougon, surgeon to the Duke de Berry; then contested for the place of "*Aide d'Anatomie*" to the faculty, and was successful. He now delivered regular courses of lectures on Surgery and Midwifery, which were successful and profitable. In May, 1823, he was examined for a degree; his Thesis on the occasion was highly spoken of, and was published. Shortly after he was appointed "Chef de Clinique" in the *Hôpital de l'Ecole*, under Dr. Bougon. In 1824, he entered the School of Medicine as Professor Agrégé. In 1828, he was unanimously chosen Surgeon to the Bureau Central, and was immediately appointed to the hospital *St. Antoine* in the quality of Surgeon-in-Chief *ad interim.*

"Though always industrious, this seems to have been the period when he devoted himself most intensely to study, and then it was that he was occupied in preparing the numbers of articles which he shortly afterwards began to publish. * *

"After remaining for a short time at St. Antoine, he was transferred to the *Hôpital de la Pitié*, where he had to himself a separate and wholly independent service. Whilst at *la Pitié*, his lectures were continued. One more promotion brought him from this latter hospital to that of *la Charité*, where he has remained ever since, followed in his morning visits by a larger number of pupils than any other surgeon in Paris."

With his career as surgeon to *la Charité* the reader is familiar.

The history of Velpeau is the history of all. With few exceptions, these distinguished men have risen from the lower ranks of society, have absolutely fought their way from poverty and obscurity to wealth and high-station. It certainly speaks much for a system that thus leaves an avenue to intellect and industry. There is something, moreover, in the contemplation of these successes that kindles the brain and nerves the arm, that makes the eye brighten and the heart glow. But is there not another side to this picture? It does not tell of the many fine and gentle natures that gave way; of the many delicate spirits that fell crushed and broken in the strife; of the high hearts that went down in despair, after toiling morn and night until the book or pen

> "Vibrated, as the ever-beating heart
> Shook the weak hand that grasped it."

No, no, it does not tell any of this—and perhaps it is well.

